# Geographic Disparities of Alzheimer’s Disease Mortality in Females With Breast Cancer

**DOI:** 10.1093/geroni/igab046.229

**Published:** 2021-12-17

**Authors:** Igor Akushevich, Arseniy Yashkin, Julia Kravchenko

**Affiliations:** 1 Duke University, Durham, North Carolina, United States; 2 Duke University, Morrisville, North Carolina, United States

## Abstract

Our estimates showed significant gaps in mortality rates between the West and East parts of the U.S. when these rates are based on death certificate data. These geographic disparities were persistent over time and could not be fully explained by differences in demographic and socioeconomic characteristics, comorbidities, and/or differences in AD coding between these regions. However, incidence and incidence-based mortality rates based on Medicare data do not reproduce these geographic disparities. Death certificate-based patterns hold for the subset of the population with breast cancer, e.g., for subpopulation for which breast cancer was listed as a secondary cause of death. Therefore, SEER-Medicare data, which contains both death-certificate records and Medicare administrative claims for the same individuals can be used to resolve this inconsistency in findings. Analysis of breast cancer patients from two SEER registries in NJ and WA states in SEER-Medicare data (2000-2013) showed that the fraction of deceased individuals with an underlying cause AD among those who had a Medicare diagnosis of AD is 2.5-3.5 times (depending on the Medicare ascertainment algorithm) higher in WA comparing to NJ (p<0.0001). The odds ratio of not-having AD as an underlying cause is 1.3 for WA vs. NJ and increases with age, for non-white races, and unmarried individuals. Our findings do not support the hypothesis of higher rates of AD in WA state but show that AD is likely underrepresented in death certificate in NJ and possibly other East coast states.

